# THE IMPACT OF A DIGITAL CANCER SURVIVORSHIP PATIENT ENGAGEMENT TOOLKIT ON OLDER CANCER SURVIVORS’ HEALTH OUTCOMES

**DOI:** 10.1093/geroni/igac059.2736

**Published:** 2022-12-20

**Authors:** Eun-Shim Nahm, Mary McQuaige, Katarina Steacy, Nancy Corbit, Shijun Zhu, Hohyun Seong

**Affiliations:** University of Maryland School of Nursing, Baltimore, Maryland, United States; University of Maryland Marlene and Stewart Greenbaum Comprehensive Cancer Center, Baltimore, Maryland, United States; University of Maryland Marlene and Stewart Greenbaum Comprehensive Cancer Center, Baltimore, Maryland, United States; University of Maryland Marlene and Stewart Greenbaum Comprehensive Cancer Center, Baltimore, Maryland, United States; University of Maryland School of Nursing, Baltimore, Maryland, United States; University of Maryland School of Nursing, Baltimore, Maryland, United States

## Abstract

Cancer is a disease that predominantly affects older adults. The median age at diagnosis is 66 years and 62% of the 15.5 million American cancer survivors are age ≥65 years. Provision of supportive care after treatment is critical to this group due to their complex care needs; however, limited resources are available to them. As increasing numbers of older survivors adopt technology, digital health programs have significant potential to help them improve their health and communicate with their providers. Previously, we developed/tested a digital Cancer Survivorship Patient Engagement Toolkit program for older adults, CaS-PET Silver. The aim was to examine the preliminary impact of CaS-PET Silver on older survivors’ health outcomes. This was a 2-armed RCT with two observations (baseline, 8 weeks) on a sample of 60 survivors age ≥65 years (mean age, 70.1±3.8), who were treated with curative intent within 12 months from enrollment (02/2020-01/2022, COVID-19 pandemic). Outcomes included health-related quality of life (HRQoL), self-efficacy for coping with cancer, symptom burden, health behaviors, and patient-provider communication. Data were analyzed using descriptive statistics, linear mixed models, and content analysis. The majority of participants were black (68.3%, n=41) and female (56.6%, n=34). At 8 weeks, CaS-PET Silver group showed significantly improved physical HRQoL (p < .001, ES=0.64) and symptom burden (p=.053, ES= -0.41). Self-efficacy (ES=0.56), mental HRQoL (ES=0.26), and communication (ES=0.40) showed a tendency to improve. Most participants reported benefits from the program on health management (mean, 19.41±2.6 [3–21]). Further research is needed with larger, diverse older cancer populations.

